# Microfluidic Paper-Based Device Incorporated with Silica Nanoparticles for Iodide Quantification in Marine Source Dietary Supplements

**DOI:** 10.3390/s24031024

**Published:** 2024-02-05

**Authors:** Mafalda G. Pereira, Ana Machado, Andreia Leite, Maria Rangel, Adriano Bordalo, António O. S. S. Rangel, Raquel B. R. Mesquita

**Affiliations:** 1 CBQF—Centro de Biotecnologia e Química Fina, Laboratório Associado, Escola Superior de Biotecnologia, Universidade Católica Portuguesa, Rua Diogo Botelho 1327, 4169-005 Porto, Portugal; magpereira@ucp.pt (M.G.P.); arangel@ucp.pt (A.O.S.S.R.); 2 ICBAS—Instituto de Ciências Biomédicas Abel Salazar, University of Porto, Rua Jorge Viterbo Ferreira 228, 4050-313 Porto, Portugal; ammachado@icbas.up.pt (A.M.); bordalo@icbas.up.pt (A.B.); 3 CIIMAR—Interdisciplinary Centre of Marine and Environmental Research, University of Porto, Novo Edifício do Terminal de Cruzeiros do Porto de Leixões, Avenida General Norton de Matos, S/N, 4450-208 Matosinhos, Portugal; 4 REQUIMTE-LAQV, Departamento de Química e Bioquímica, Faculdade de Ciências, Universidade do Porto, Rua do Campo Alegre, 4169-007 Porto, Portugal; 5 REQUIMTE-LAQV, Instituto de Ciências Biomédicas de Abel Salazar, Universidade do Porto, Rua Jorge Viterbo, 4050-313 Porto, Portugal; mrangel@icbas.up.pt

**Keywords:** iodide determination, µPAD, TMB reaction, food samples, dietary supplements

## Abstract

Iodine is an essential micronutrient for humans due to its fundamental role in the biosynthesis of thyroid hormones. As a key parameter to assess health conditions, iodine intake needs to be monitored to ascertain and prevent iodine deficiency. Iodine is available from various food sources (such as seaweed, fish, and seafood, among others) and dietary supplements (multivitamins or mineral supplements). In this work, a microfluidic paper-based analytical device (μPAD) to quantify iodide in seaweed and dietary supplements is described. The developed μPAD is a small microfluidic device that emerges as quite relevant in terms of its analytical capacity. The quantification of iodide is based on the oxidation of 3,3′,5,5′-tetramethylbenzidine (TMB) by hydrogen peroxide in the presence of iodine, which acts as the catalyst to produce the blue form of TMB. Additionally, powder silica was used to intensify and uniformize the colour of the obtained product. Following optimization, the developed μPAD enabled iodide quantification within the range of 10–100 µM, with a detection limit of 3 µM, and was successfully applied to seaweeds and dietary supplements. The device represents a valuable tool for point-of-care analysis, can be used by untrained personnel at home, and is easily disposable, low-cost, and user-friendly.

## 1. Introduction

Iodine is an essential nutrient for humans, and it is naturally occurring or added to food [1]. Due to its crucial role in producing thyroid hormones, iodine is a key parameter in assessing the public health condition [2]. The typical diseases caused by impaired function of the thyroid, prompted by insufficient iodine, are classified as iodine deficiency disorders (IDD) [3]. For pregnant women, low levels of this analyte can cause spontaneous abortion, stillbirth, and congenital anomalies [4], so it is possible to conclude that iodine deficiency is one of the most preventable causes of brain damage worldwide [5]. The ocean is the main reservoir worldwide of iodine, but it is usually found in soil, seawater, and seaweed as iodide [6]. The iodine content in most foods is generally low, but bread, milk, and marine-origin foods, namely seaweed, are the major sources of iodine in a typical human diet [3]. The World Health Organization (WHO), the United Nations Children’s Fund (UNICEF), and the International Council for the Control of Iodine Deficiency Disorders Global Network (ICCIDD) recommended salt iodization as the most cost-effective solution to attempt to reduce and prevent IDD. However, this approach is based on the internal policies of each country and can lead to cardiovascular diseases and high blood pressure conditions if salt is consumed at exceeding levels. Despite the possible fortification of foodstuffs, pregnant women and children under two years of age may require additional introduction of dietary supplements to achieve the required iodine levels recommended by the WHO [2].

The methods currently used to analyse the iodine content of foodstuffs, plants, soil, or biological samples are relatively expensive and not easily acquired by many laboratories [7]. One of the most known methods is based on the Sandell–Kolthoff reaction, which requires a digestion step where the possible interferences are eliminated, followed by a two-step slow reaction [8]. Moreover, it uses carcinogenic and toxic reagents [9]. Over the years, new methods have been developed [10,11,12,13,14,15,16,17,18,19] with new types of equipment and techniques that can efficiently determine iodine or iodide across a spectrum of sample matrices, namely in food [12,13,14,16,17,19] and biological samples [10,11,13,15,18].

Nowadays, people are becoming more self-aware of health, so new diagnosis tools that are simple, effective, and enable rapid testing have gained high relevancy [20,21]. According to the ASSURED criteria established by the WHO, these new tools must be “affordable, sensitive, specific, user-friendly, rapid and robust, equipment-free, and derivable to end-users” [22]. In this context, paper-based devices can be an effective approach, exploiting advantages such as usage by untrained personnel and being equipment-free (not needing any kind of exterior power source) combined with low reagents and sample consumption [23]. The microfluidic paper-based analytical device (µPAD) is a recently described paper platform, where channels are defined with millimetric dimensions, and two different zones are established, a hydrophobic and a hydrophilic zone [24]. On a colourimetric paper device, the detection process can be qualitative, providing only a yes or no response; semi-quantitative, by observing the colour gradient; or quantitative, by analysing the colour with computer software [21]. An accurate quantitative analysis requires a pre-established calibration curve obtained from the image analysis, which can be performed with a scanner, followed by software treatment, where the RGB data are used to set different intensity values of the coloured product [25]. Regarding the determination of iodide, one paper was reported [19], but it employs wax printing technology and needs a personalized smartphone holder for signal acquisition.

This work aimed to devise a µPAD for quantifying iodide in different sample types based on the reaction between TMB and hydrogen peroxide, coupled with an environmentally friendly assembly technique. In our laboratory, we have developed a new assembly method, without the use of wax printing, by stacking paper discs that have the reagent either on the top or bottom layer [24,26]. The chosen reaction between 3,3′,5,5′-tetramethylbenzidine (TMB) and hydrogen peroxide has been detailed in the work of Lin et al. [27]. The TMB is a colourless reagent which turns blue in the presence of hydrogen peroxide due to its oxidation. As this reaction is very slow, iodide was used as a catalyser. In the end, the developed device would help the end-user to analyse iodide in dietary supplements from marine sources, without any pretreatment, which is almost mandatory in most traditional methods of assessing iodide intake.

## 2. Material and Methods

### 2.1. Reagents and Solutions

All solutions used in this work were prepared with analytical grade chemicals and Milli-Q water (resistivity > 18.2 MΩ/cm, Millipore, Burlington, MA, USA).

A 0.1 M iodide stock solution (Hanna HI4011-01) was used to prepare two intermediate iodide standard solutions of 5 mM and 0.250 mM weekly. From the 0.250 mM iodide solution, the working standards from 10 to 100 μM were also prepared weekly.

The 0.8 M acetic acid solution was prepared by diluting 4.6 mL of acetic acid (d = 1.05, 100% glacial, Merck, Darmstadt, Germany) in 100 mL of water. Then, the pH was adjusted to 3.6 with 2.5 M NaOH, as reported by Lin et al. [27].

The 5 M hydrogen peroxide solution was prepared by dilution of the commercial solution (d = 1.11, 30% Merck, Darmstadt, Germany).

The solution of hydrogen peroxide and acetic acid was prepared daily by combining 240 μL of 0.8 M acetic acid and 10 μL of 5 M hydrogen peroxide.

The 3,3’,5,5’-tetramethylbenzidine (TMB) solution was prepared by dissolving 24 mg of TMB (Sigma-Aldrich, Saint Louis, MO, USA) in 50 mL of ethanol (96%, Labchem, Greenford, England) and then adding 50 mL of water, resulting in a 1 mM concentration, as described by Palladino et al. [28]. This solution was stored in a dark bottle and refrigerated.

A silica powder suspension was prepared by mixing 15 mg of the powder in 5 mL of water, as described for other solid components by Ferreira et al. [29]. This suspension was used for every 2 μPADs. The preparation of the silica powder is detailed in the Section Preparation of Silica Powder.

#### Preparation of Silica Powder

The silica used in this work was obtained from rice husks (RHs) from *Oryza sativa* L. kindly supplied by Novarroz—Produtos Alimentares, S.A. (Oliveira de Azemeis, Portugal). The RHs were washed with water to remove soil and dust. Subsequently, the material was oven-dried at 80 °C overnight. Then, the RHs were calcinated at 600 °C for 4 h with a ramp of 5 °C min^−1^, resulting in the production of rice husk ash (RHA) under controlled conditions. Afterwards, 16.6 g of RHA was combined with 15 g of citric acid and 200 mL of water. The mixture was stirred in a MARS 6 microwave (CEM Corporation) for 30 min at 50 °C. The resulting blend was filtered, and the treated rice husk ash (TRHA) was placed in an oven until completely dried.

### 2.2. µPAD Assembly

**Figure 1:**
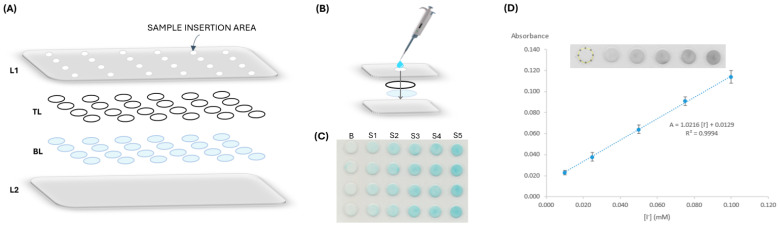
Iodide µPAD assembly; (**A**) schematic representation of the alignment; L1, laminating pouch containing the sampling holes; TL, top layer containing silica powder, hydrogen peroxide, and acetic acid; BL, bottom layer containing TMB; L2, laminating pouch; (**B**) representation of a single reading unit, where sample loading is 20 µL; (**C**) image scan of the device for a calibration curve, where S_i_ represents the different iodide standards and B is the blank; (**D**) software RGB-treated image (Image J, version 1.53) for intensity measurements and absorbance calculation, with the respective calibration curve plotting, where the dashed circle represents the selected area for intensity counts.

The μPAD assembly consists of establishing a hydrophilic detection area for the reaction to occur and a hydrophobic area to isolate the independent detection areas. To set the hydrophobic area, a 75 × 110 × 0.125 mm plastic laminating pouch (Q-Connect, Gent, Belgium) was perforated with twenty-four 3 mm holes for sample insertion in a 6 columns × 4 rows arrangement. The hydrophilic area consisted of twenty-four reading units established with the stacking of two filter paper discs aligned with the holes of the plastic laminating pouches (Figure 1A).

For the iodide determination, two different types of Whatman filter paper were used, both with a 9.5 mm diameter, obtained by using a paper puncher (3/8″ EK tools). The top layer (TL in Figure 1A) consisted of Whatman Grade 4 (W4) filter paper discs, which were soaked in the silica suspension solution for 30 s under manual agitation and then placed in the oven to dry for 30 min at 50 °C. After that, these discs were loaded with 10 μL of the acetic acid and hydrogen peroxide solution and were set to dry in the oven at 50 °C for 10 min. The colour reagent layer, the bottom layer (BL in Figure 1A), was prepared by loading 10 μL of TMB and was left to air dry at room temperature for 20 min.

Following these procedures, the two-disc units were aligned with the sample hole in the plastic pouch and sealed through the laminator (A3-330C High Quality Laminator), establishing the twenty-four reading units with the two distinctive hydrophobic and hydrophilic areas.

### 2.3. Iodide Determination and Data Processing

For the determination of iodide, 20 μL of standard/sample was inserted through the sample hole of each reading unit (Figure 1B). After waiting 25 min for the colour to develop, the μPAD was scanned (EPSON Stylus SX100, Los Alamitos, CA, USA) on the colour reagent side, bottom sheet of the laminating pouch (L2 in Figure 1A). To analyse the colour intensity of each reading unit, a free image processing software was used (ImageJ), and the intensity value was obtained using RGB (red, green, blue) filters. The red filter was selected as the complementary colour of the formed product (Figure 1C). The absorbance was calculated using the formula: A = log (*I*_0_/*I*), where A is the absorbance value, I is the mean measured intensity of the standard/sample (#4 replicas), and I_0_ is the mean measured intensity of the blank (#4 replicas) obtained by loading water. A calibration curve was established correlating the calculated absorbance values and the iodide standard concentration (Figure 1D), resulting in the quantification of iodide in samples by interpolation.

### 2.4. Sample Preparation

Two types of dietary supplements were used as samples, iodide pharmaceuticals and edible algae, and were purchased in a local supermarket. The acquired iodide pharmaceutical tablets were crushed in a mortar and dissolved in 25 mL of water. All algae samples were immersed in water (volumes of 25 or 300 mL) for about 10 min. Regarding the dry algae (dried seaweed), they were previously washed and blended before immersing them in water; the wet algae sample (sea cabbage) was washed and then immersed in water.

For all the samples, after dissolution/immersion in water, the extract was filtered before loading it into the developed device. Details and ID of the samples can be found in the Supplementary Materials (Table S1).

### 2.5. Accuracy Assessment—Comparison Method

To validate the developed method, a comparison was made between the paper device and the ion-selective electrode method using an iodide combined electrode (Mettler Toledo perfectION combination, DX series half-cells) connected to a potentiometer (Crison GLP 21) for signal measurement.

To perform the potentiometric method, iodide standard solutions in the range of 5 × 10^−6^ to 5 × 10^−3^ M were prepared, and the analyte concentrations were calculated by interpolation in the corresponding calibration curve: E = f (log [I^−^]). For each measurement, 5 mL of standard/sample was mixed with 5 mL of the ionic strength-adjusting solution (0.4 M KNO_3_).

## 3. Results and Discussion

### 3.1. Preliminary Studies

As mentioned above, the reaction between TMB, hydrogen peroxide, and iodide in acetic acid medium had been previously reported by Lin et al. [27] as an in vitro assay with all the solutions added separately. Therefore, before testing the reaction performance in a paper platform, a batchwise study was carried out aiming to restrict the use to only two reagent solutions, which would simplify the paper-based approach. In this context, a calibration curve was established using two reagent solutions: a mixture of acetic acid and hydrogen peroxide, and the TMB reagent solution. This calibration curve was compared with a calibration curve established using the three reagent solutions separately. For both calibration curves, the iodide standards were added last. The calibration curves resulted in similar slopes and intercepts, indicating that there was no influence when using acetic acid and hydrogen peroxide as a mixture. Therefore, it became feasible to design a µPAD with two paper layers to ensure a vertical flow approach; initially, a Whatman 1 filter paper was used with the top layer loaded with the solution of acetic acid and hydrogen peroxide and the bottom layer loaded with colour reagent, TMB. The µPAD was scanned on the colour reagent side.

### 3.2. µPAD Design

#### 3.2.1. Study of the Filter Paper Type

**Figure 2:**
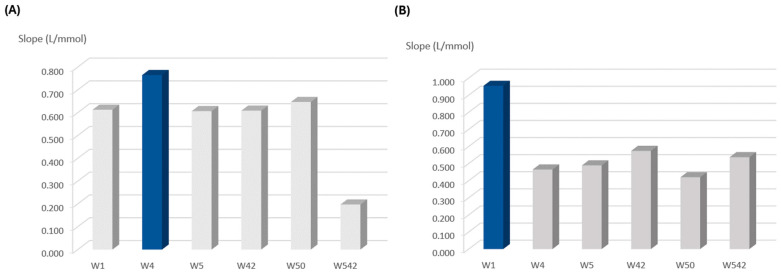
Influence of filter paper porosity and treatment on the calibration curve slope (sensitivity): (**A**) studies for the top layer to be loaded with hydrogen peroxide and acetic acid; (**B**) studies for the bottom layer to be loaded with TMB reagent; the dark blue column represents the chosen option.

The type of filter paper on which the reagents were loaded was evaluated by assessing its influence on the calibration curve. First, a test was performed for the top layer paper disc, which consisted of testing different filter papers (Figure 2A). For that, different pore sizes of qualitative paper were tested: 20–25 μm, Whatman 4 (W4); 10–20 μm, Whatman 1 (W1); and 2.5–5 μm, Whatman 5 (W5). Then, filter paper with the same porosity (2.5–5 μm) with different treatments was also tested: qualitative (W5); ashless, Whatman 42 (W42); hardened, Whatman 50 (W50); and hardened ashless Whatman 542 (W542).

The calibration with the highest slope, providing the highest sensitivity, corresponded to the one with W4 filter paper (Figure 2A). Therefore, this filter paper (W4) was chosen for the top layer to be loaded with the solution of hydrogen peroxide and acetic acid.

For the bottom layer, the same filter papers as for the top layer were tested, and the results showed that W1 filter paper provided the highest calibration curve slope and thus, the higher reaction sensitivity (Figure 2B).

#### 3.2.2. Influence of TMB Concentration

Following the choice of paper, the influence of the concentration of the TMB on the reaction sensitivity was studied.

**Figure 3:**
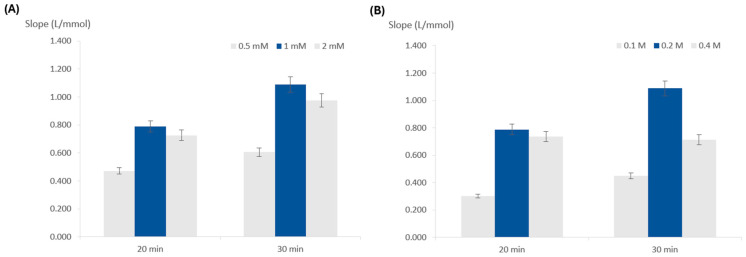
Influence of the reagent concentrations on calibration curve slope (sensitivity); (**A**) TMB concentration; (**B**) hydrogen peroxide concentration; the dark blue columns represent the chosen option and the error bars represent 5% deviation.

For this purpose, three concentrations were tested, 0.5, 1, and 2 mM, keeping the remaining composition unchanged (Figure 3A). The highest slope was obtained for the 1 mM concentration, meaning this was the concentration providing the best sensitivity, so it was chosen.

#### 3.2.3. Influence of Peroxide Concentration

To study the influence of the hydrogen peroxide concentration, concentrations of 0.1, 0.2, and 0.4 M were assessed (Figure 3B). The calibration curve with the highest slope corresponded to 0.2 M, so this was chosen for further testing.

The acetic acid was used only for pH adjustment, so its concentration was not tested, and 0.8 M was used throughout the work.

#### 3.2.4. Order of Layers

**Figure 4:**
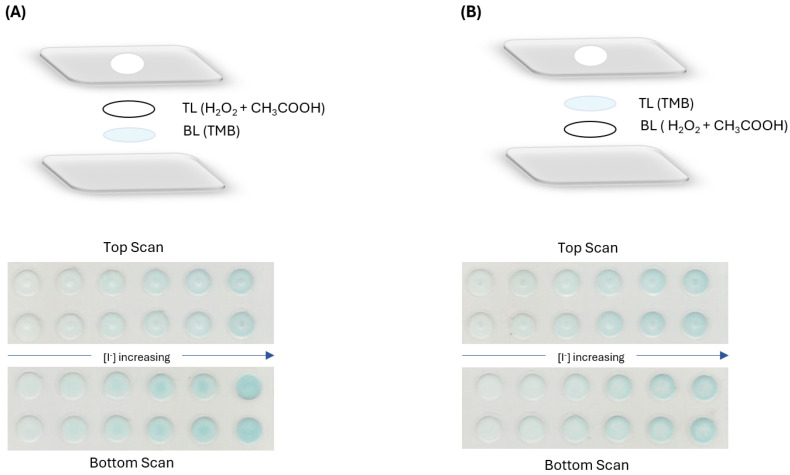
Tested configurations with respective image scan on both scanning sides, (**A**) H_2_O_2_ and CH_3_COOH in the top layer and TMB in the bottom layer; (**B**) TMB in the top layer with H_2_O_2_ and CH_3_COOH in the bottom layer.

After setting the type of paper to be used and the reagent concentrations, the influence of the order of the layers in the determination sensitivity was tested. Calibration curves were established with two configurations: the H_2_O_2_ and CH_3_COOH in the top layer and TMB in the bottom layer (Figure 4A) and the reverse order with TMB in the top layer and H_2_O_2_ and CH_3_COOH in the bottom layer (Figure 4B).

For both configurations, the device was scanned on the top side and the bottom side (Figure 4). The initial configuration (Figure 4A), with the colour reagent in the bottom layer, and scanning the bottom layer was the combination that enabled a higher sensitivity, so it was kept.

#### 3.2.5. Sample Volume

The influence of the standard/sample volume in the determination of sensitivity was evaluated and volumes of 15, 20, and 25 μL were used in the previously set design to establish calibration curves (Figure S1). For each volume, a scan was made at 20, 25, and 30 min to ascertain if higher volumes eventually needed more reaction time. For all periods analysed, it was possible to conclude that 20 μL was the volume that resulted in higher sensitivity, so it was chosen as the standard/sample volume.

#### 3.2.6. Time to Scan

Following the previous study, the time between loading the sample/standard and scanning the device, named time to scan, was evaluated. From the in vitro studies, it was possible to conclude that the absorbance reading should be carried out 30 min after the beginning of the reaction. However, because the dynamic of the reaction in the paper is different, several waiting times were studied, from 10 min to 30 min, with 5 min intervals (Figure S2). Therefore, it was possible to conclude that the best sensitivity was obtained at 25 and 30 min. Since there were no differences (relative deviation of the calibration curve slopes ≈ 1%), 25 min was chosen, ensuring a main feature of the μPAD was a rapid response.

#### 3.2.7. Use of Silica for Improving Repeatability

The colour uniformity in a paper-based device is always a challenge and usually requires the placement of the colour reagent on top to narrow down the reading area [24]. However, for this determination, that proved not to be the best approach, as shown in Section 3.2.4. Therefore, aiming to improve the colour product uniformity, an additional study was performed based on the method described by Evans et al. [30] that reported the use of silica nanoparticles in a microfluidic device to improve colour intensity and uniformity. To test this hypothesis, calibration curves were established using devices with and without silica particles on the top layer (Figure S3).

Incorporating the silica particles promoted the colour product uniformity, evaluated by calculation of the relative standard deviation of the slope (RSD), as the slope RSD of the calibration curve with silica is 9% and without silica is 17%. Additionally, the incorporation of the silica nanoparticles also increased the absorbance signal and the calibration curve slope itself, by about 30%, improving the µPAD sensitivity.

#### 3.2.8. Influence of pH

Although the optimum pH reported for this reaction is 3.6 [27], a study of the influence of the reaction pH was performed, where four different pH values were tested by adjusting the acetic acid solution pH. The pH 3.6 was chosen from the tested pHs of 1.1, 2.3, 3.6, and 4.7 (Figure S4), as it provided the highest calibration curve slope.

### 3.3. Assessment of Matrix Potential Interferences

Aiming to apply this device for iodide quantification in marine source dietary supplements, the potential matrix interference was assessed by testing synthetic preparations. Three iodine standards of 50 µM were prepared, one in water, one in a solution mimicking the composition of some dietary supplements (multivitamin mix), and one in a 40 g/L sea salt solution (Sigma S9883). The absorbance signal of the standard prepared in each matrix was compared to the absorbance standard prepared in water, and the relative deviation was calculated (Table 1).

The results allowed us to conclude that there were no significant interferences for the tested matrixes.

### 3.4. Stability Studies

#### 3.4.1. Stability of the Reaction Product

Having established the operation conditions, it was important to assess the stability of the formed coloured product. After inserting the standard/sample in the μPAD, it was important to assess how long the formed coloured product was stable. Thus, a calibration curve was prepared, and the µPAD scanned at different intervals, up to 120 min. The obtained calibration curve slopes (sensitivity) for each scanning time were compared (Figure S5). Although the calibration curve slope increased until 45 min, an increment in the intercept (about 35%) could also be noted, and the linearity was significantly reduced (about 97%). Consequently, it was shown that it is possible to obtain sensitive results at 25 min and that the µPAD could be scanned up to 40 min after inserting the sample without linearity loss.

#### 3.4.2. µPAD Stability

It was also crucial to know the stability of the device itself before introducing the standard/sample. In this way, the storage time before its use was evaluated.

**Figure 5:**
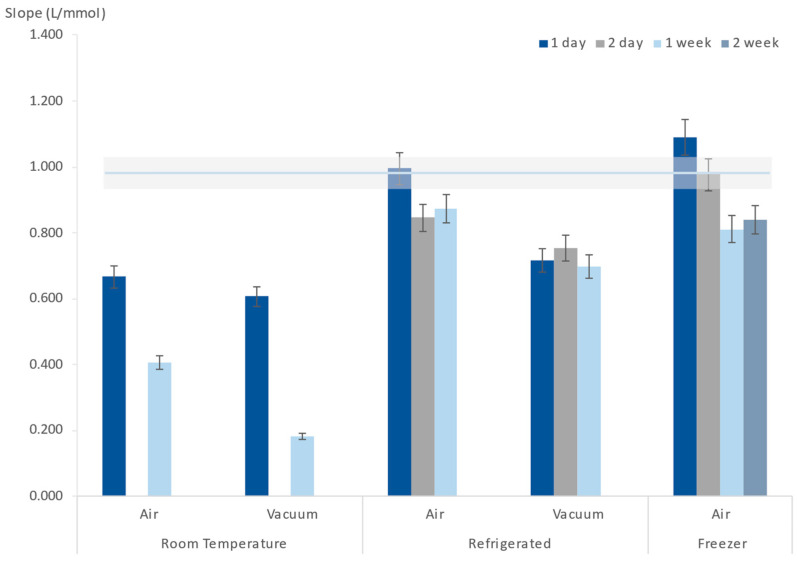
Influence of μPAD storage conditions (temperature and presence of air) on the calibration curve slope (sensitivity); the horizontal line represents the calibration curve average slope of the freshly prepared devices, and the shadow area represents a 5% deviation of that average.

Different storage conditions were tested: stored at room temperature, in the refrigerator (about 4 °C), and in the freezer (about −8 °C); stored in the presence of air or in vacuum. In all tested storage conditions, the μPADs were protected from light by wrapping them in aluminium foil. The storage periods varied from 1 day to 2 weeks (Figure 5). After each storage period, iodide standards were inserted, as they normally would be, and the obtained results were compared with a freshly prepared μPAD to ascertain if there were noticeable differences in sensitivity (Figure 5).

It could be perceived that vacuum and room temperature were not adequate storage conditions for the μPADs because even after just one day, a significant decrease in the calibration curve slope could already be observed.

After 1 day of refrigerated storage (Figure 5), the device sensitivity was maintained, resulting in a calibration curve slope not different from a freshly prepared device. Therefore, an even lower storage temperature was tested, and when stored in the freezer, a two-day-old μPAD still produced a calibration curve similar to the one obtained from a freshly prepared device (Figure 5).

### 3.5. Features of the µPAD

Having established the best operation conditions for the determination of iodide in a microfluidic device, the features of the developed µPAD were summarized in Table 2. The limit of detection (LOD) and the limit of quantification (LOQ) were calculated as the concentration corresponding to three and ten times, respectively, the standard deviation of the calibration curve intercept (*n* = 5), following IUPAC recommendations [31].

The interday and intraday repeatability was assessed by calculating the relative standard deviation (RSD) of the calibration curve slope (*n* = 3). The reagent consumption per device was calculated accounting for 24 reading units and the reagent volume and concentration required for preparing that number of units.

The selectivity of the device for this ionic form of iodine, iodide, was tested by preparing iodate standards and loading them in the developed µPAD. All the standards up to 100 µM of iodate resulted in absorbance values within the standard deviation of the iodide calibration curve intercept, thus indicating no significant response for iodate.

### 3.6. Accuracy Assessment—Application to Samples

The accuracy assessment was performed by analysing several supplement samples, both pharmaceuticals and edible algae, with the developed μPAD ([Iodide]_μPAD_) and comparing the results with those obtained by the potentiometric method using an ion-selective electrode ([Iodide]_ISE_). Once the iodide content was determined for each sample by both methods, the relative error (RE) was calculated: RE % = (([Iodide]_μPAD_ − [Iodide]_ISE_)/[Iodide]_ISE_) (Table 3).

A linear relationship was established, and the following equation was obtained [Iodide]_μPAD_ = 0.955 (±1.223) × [Iodide]_ISE_ − 0.581 (±32.626), where the values in brackets correspond to the 95% confidence interval.

It was possible to conclude that there was no evidence for significant differences between the two sets of results [32] as the obtained slope was not statistically different (ρ > 0.05) from 1 and the intercept was close to 0, with a 95% confidence interval.

## 4. Conclusions

In this work, a new microfluidic paper-based analytical device, µPAD, for iodide determination in dietary supplements was devised. This new method proved to be sensitive to the required iodide levels and an innovative, affordable platform that results in a rapid, robust, and user-friendly analysis. The incorporation of silica nanoparticles obtained from rice husks not only improved the device sensitivity but also featured a sustainable approach (circular economy).

Several supplements from marine sources were successfully analysed with the µPAD in the range of 10 to 100 µM with a limit of detection and quantification of 3.0 and 10 µM, respectively. The developed μPAD could be a suitable alternative for iodide determination when compared to currently used methods for iodine/iodide determination (Table 4). In fact, it presents a favourable combination of limit of detection and analysis time. With a cost of consumables of EUR 0.12 per device, this platform has been shown to be simpler, less laborious, and more environmentally friendly than the most employed method for measuring this analyte, the Sandel–Kolthoff method [10].

To this day, only one similar platform has been developed [19]; however, it requires a personalized holder for the smartphone-assisted determination, the µPAD fabrication involves wax printing, which could raise some sustainability problems, and the detection limit is not as low as the one in this work.

The developed device is affordable and sustainable due to the low quantities of reagents and samples required. The potential drawback could be that the detection was based on a flatbed scanner instead of a smartphone-assisted determination, as in some recently reported methods [19,33]. This device offers a simpler alternative to accessible testing since it allows easy-to-interpret results and is easy to use, in line with the new trend of point-of-care analysis [34,35]. Regardless, this device serves as a valuable tool and can be used by individuals without specialized training, making it suitable for home use due to its ease of interpretation, disposable nature, user-friendly features, and affordability.

## Figures and Tables

**Table 1:** Study of potential inference from marine source dietary supplements matrix in the calculated absorbance; RD, relative deviation obtained by comparing the absorbance of 50 µM iodide standard in the different synthetic matrixes to a water matrix.

Synthetic Matrix	Tested Compounds and Concentrations	RD
Multivitamin mix	Calcium 3.71 mg/L	4%
	Magnesium 0.78 mg/L	
	Potassium 540 mg/L	
	Sodium 2.31 g/L	
	Chloride 6.57 mg/L	
	Lactic acid 0.10 g/L	
	Citric acid 0.40 g/L	
	Glucose 0.50 g/L	
Sea salts (Sigma S9883)	Calcium 400 mg/L	−6%
	Magnesium 1.32 g/L	
	Potassium 350 mg/L	
	Sodium 10.7 g/L	
	Chloride 19.5 g/L	
	Carbonate 170 mg/L	
	Boron 5.6 mg/L	
	Stroncium 8.8 mg/L	

**Table 2:** Features of the developed μPAD for iodide determination; LOD, limit of detection; LOQ, limit of quantification; RSD, relative standard deviation.

Dynamic range	10–100 µM
Typical calibration curve (A = slope ± SD × [Iodide] mM + intercept ± SD)	A = 1.03 ± 0.13 × [Iodide] + 0.010 ± 0.001
LOD	3.0 µM
LOQ	10 µM
Intraday repeatability, slope RSD ^a^	3%
Interday repeatability, slope RSD ^a^	3%
Time to scan	25 min
Reagent consumption/μPAD	15 mg silica powder 1.7 mg H_2_O_2_ 11 mg CH_3_COOH 58 mg TMB

^a^ *n* = 3.

**Table 3:** Accuracy assessment by comparing the results obtained with the developed µPAD ([Iodide]_μPAD_) and the potentiometric method using an ion-selective electrode ([Iodide]_ISE_) by calculating the relative error (RE) percentage; SD, standard deviation.

Sample ID	[Iodide]_ISE_ ± SD, µM	[Iodide]_μPAD_ ± SD, µM	RE, %
#Pharm 1	49.9 ± 2.8	47.4 ± 4.3	−5.0
#Pharm 2	34.3 ± 1.5	31.6 ± 1.1	−8.0
#Pharm 3	82.1 ± 3.1	78.5 ± 1.6	−4.4
#Pharm 4	48.9 ± 2.2	45.6 ± 0.1	−6.8
#Algae 1	22.9 ± 1.4	22.6 ± 0.7	−1.3
#Algae 2	52.2 ± 0.1	50.1 ± 1.6	−4.1
#Algae 3	67.4 ± 1.9	64.2 ± 1.8	−4.7
#Algae 4	11.7 ± 0.1	12.1 ± 1.2	3.3
#Algae 5	49.5 ± 1.1	51.7 ± 0.6	4.5
#Algae 6	79.4 ± 1.8	75.5 ± 1.9	−4.9
#Algae 7	11.7 ± 1.2	12.7 ± 1.1	7.9
#Algae 8	50.7 ± 0.7	47.8 ± 1.2	−5.6
#Algae 9	80.5 ± 0.1	80.0 ± 1.3	−0.6

**Table 4:** Comparison between previously reported methods for iodine and iodide determination and the developed µPAD methodology for iodide determination; LOD, limit of detection; ICP-MS, inductively coupled plasma–mass spectrometer; ICP-OES, inductively coupled plasma–optical emission spectrometer; HPLC, high-performance liquid chromatography.

Method	Iodine Form	Sample	Analysis Time per Assay	LOD	Reference
Sandell–Kolthoff (spectrophotometry)	Iodine	Urine	1 h 30 min	<25 µg/L (0.19 µM)	[10]
ICP-MS	Iodine	Urine	<1 h 30 min	0.95 µg/L (7.5 × 10^−3^ µM)	[11]
Voltammetry	Iodide	Salt	≈1 min	0.3 mg/L (2.4 µM)	[12]
Potentiometry	Iodide, Iodate	Urine and salt	2 min	1.39 µM (iodide), 1.77 µM(iodate)	[13]
ICP-OES	Iodine	Food	5 min	0.049 mg/L (0.39 µM)	[14]
Microplate method (spectrophotometry)	Iodine	Urine	≈1 h 30 min	n.d. ^1^	[15]
Chip-based spectrofluorimetry	Iodine	Salt, pharmaceuticals, and algae	n.d. ^1^	0.028 µM	[16]
Total reflection X-ray fluorescence	Iodine	Water and dietary supplements	<15 min	180 µg/L (1.4 µM)	[17]
HPLC (spectrophotometry)	Iodide	Urine	13 min	n.d. ^1^	[18]
µPAD (spectrophotometry)	Iodide, Iodate	Seaweed	1 min	9.8 µM (iodide), 0.6 µM (iodate)	[19]
µPAD (spectrophotometry)	Iodide	Pharmaceuticals and algae	25 min	3 µM	This work

^1^ n.d.—not disclosed.

## Data Availability

The data presented in this study are available on request from the corresponding author.

## Supplementary Materials

The following supporting information can be downloaded at: https://www.mdpi.com/article/10.3390/s24031024/s1, Table S1. Details of the tested supplement samples, including identification and pretreatment; Figure S1. Influence of the sample/standard volume on the calibration curve slope (sensitivity); the error bars represent a 5% deviation, and the dark blue columns are the chosen volume; Figure S2. Influence of different times to scan on the calibration curve slope (sensitivity); the error bars represent a 5% deviation, and the dark blue column is the chosen time; Figure S3. Influence on the calibration curve slope (sensitivity) of silica nanoparticles’ incorporation in the device; the error bars represent a 5% deviation, and the dark blue column is the chosen option; Figure S4. Influence of different reaction pH values on the calibration curve slope (sensitivity); the error bars represent a 5% deviation, and the dark blue column is the chosen pH; Figure S5. Stability of the formed colour product along 120 min of reaction time by comparing the calibration curve slope (sensitivity) at the different scanning times; the dark grey bars represent the scanning time with calibration curve linearity above 0.99, and the white bars represent the scanning time with calibration curve linearity below 0.99. The dark line represents the intercept of the calibration curve for each time.
