# Supplementary material for: Microfluidic Paper-Based Device Incorporated with Silica Nanoparticles for Iodide Quantification in Marine Source Dietary Supplements
Source: Sensors (Basel). 2024 Feb 5;24(3):1024. doi: 10.3390/s24031024 (PMC10857764; doi:10.3390/s24031024)
Supplement: Supplementary file 1 [file sensors-24-01024-s001.zip › sensors-2850316-supplementary.pdf]

Supplementary Material

Microfluidic paper-based device incorporated with silica nanoparticles for iodide quantification in marine source dietary supplements

Mafalda G. Pereira[1], Ana Machado[2,3], Andreia Leite[4], Maria Rangel[5], Adriano Bordalo[2,3], António O. S. S. Rangel[1], Raquel B. R. Mesquita*[1]

[1]Universidade Católica Portuguesa, CBQF – Centro de Biotecnologia e Química Fina – Laboratório Associado, Escola Superior de Biotecnologia, Rua Diogo Botelho 1327, 4169-005 Porto, Portugal

[2]ICBAS—Instituto de Ciências Biomédicas Abel Salazar, University of Porto, 4050-313 Porto, Portugal

[3] CIIMAR – Interdisciplinary Centre of Marine and Environmental Research, University of Porto, Novo Edifício do Terminal de Cruzeiros do Porto de Leixões, Avenida General Norton de Matos, 4450-208 Matosinhos, Portugal

[4]REQUIMTE-LAQV, Departamento de Química e Bioquímica, Faculdade de Ciências, Universidade do Porto, 4169-007 Porto, Portugal

[5]REQUIMTE-LAQV, ICBAS, Universidade do Porto, 4050-313 Porto, Portugal

Corresponding author: *rmesquita@ucp.pt

Table S1. Details of the tested supplements samples, including identification and pre-treatment.

Sample Type	m (g)	Added volume of water (mL)	Sample ID	Pre-treatment	Spiked [I^-] (μM)	Filter process & type
Pharmaceutical Y200	0.1072	25	#Pharm 1	Crushed and dissolved in water	-	Vacuum Filter 595 (7μm)
			#Pharm 2		25	
Pharmaceutical Y300	0.1653	25	#Pharm 3		-	Vacuum Filter 595 (7μm)
			#Pharm 4		25	
Dried Seaweed	15.33	300	#Algae 1	Washed (400 mL), blended and then squeezed into water	25	Vacuum Filter 595 (7μm)
			#Algae 2		50	
			#Algae 3		75	
Sea Cabbage	10.324	25	#Algae 4	Immersed in water for 10 min	25	Syringe membrane Nylon (0.45 μm)
			#Algae 5		50	
			#Algae 6		75	
Sea Cabbage (washed)	10.374	25	#Algae 7	Washed (100 mL), then immersed in water for 10 min	25	Syringe membrane Nylon (0.45 μm)
			#Algae 8		50	
			#Algae 9		75	

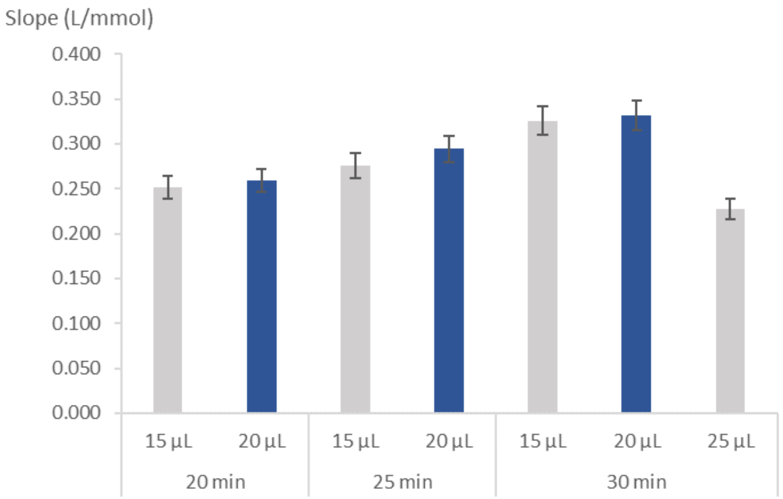

Figure S1. Influence of the sample/standard volume on the calibration curve slope (sensitivity); the error bars represent a 5% deviation and the dark blue columns the chosen volume.

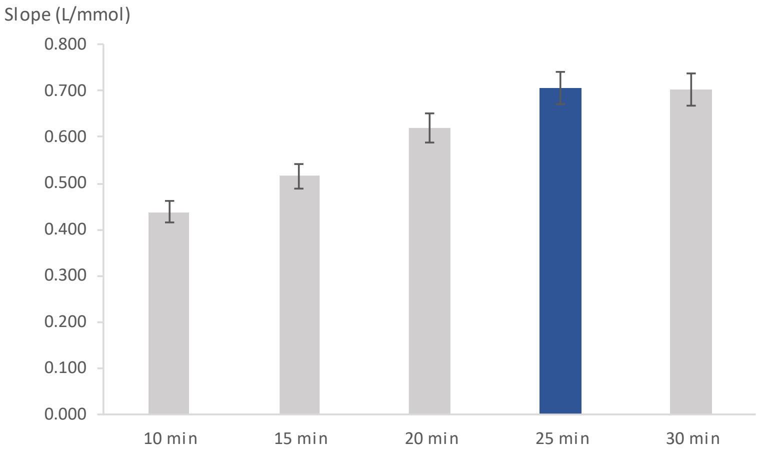

Figure S2. Influence of different time-to-scan on the calibration curve slope (sensitivity); the error bars represent 5% deviation and the dark blue columns the chosen time.

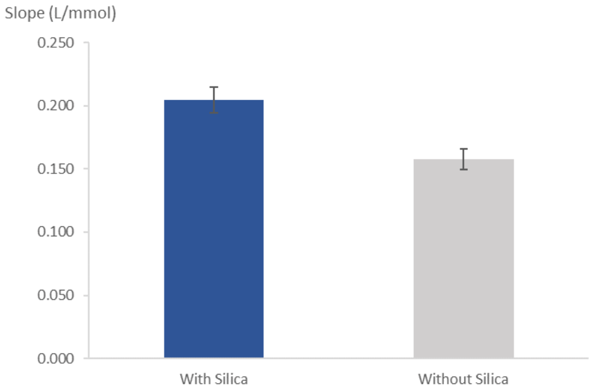

Figure S3. Influence on the calibration curve slope (sensitivity) of silica nanoparticles incorporation in the device; the error bars represent 5% deviation and the dark blue column is the chosen option.

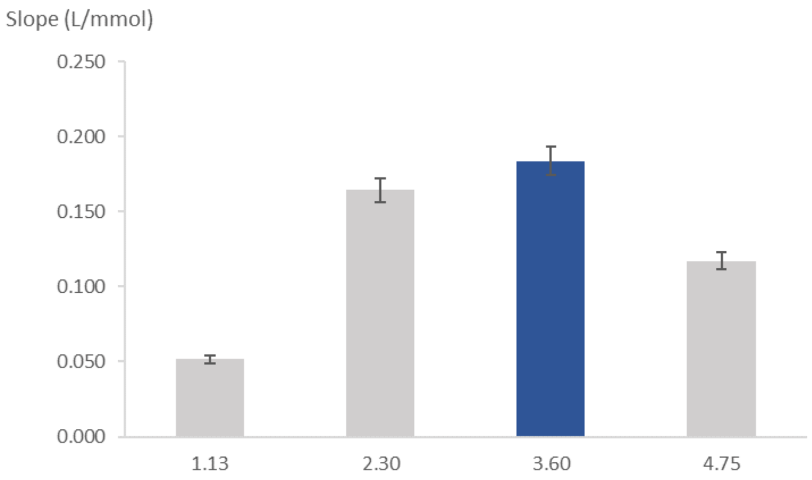

Figure S4. Influence of different reaction pH values on the calibration curve slope (sensitivity); the error bars represent a 5% deviation, and the dark blue column is the chosen pH.

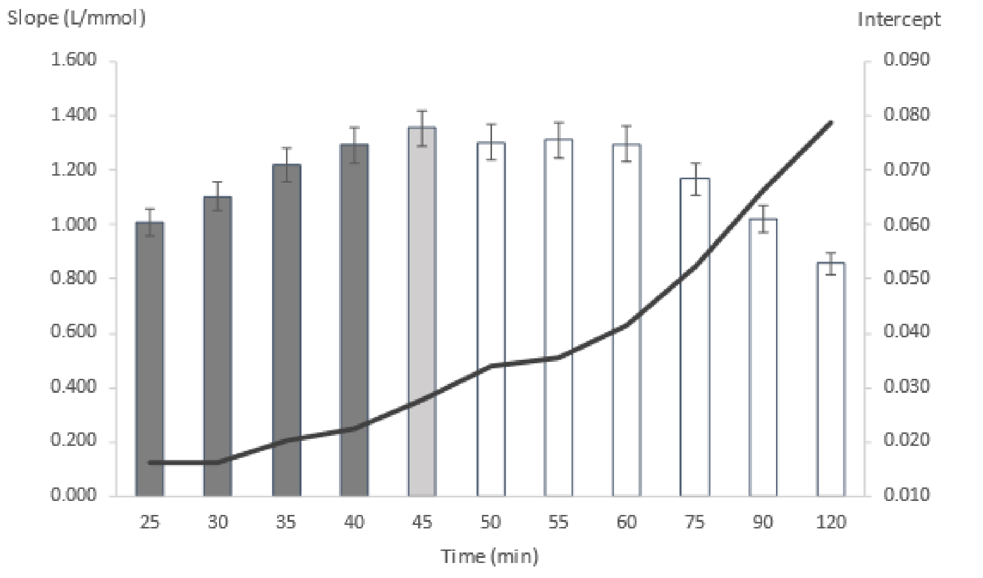

Figure S5. Stability of the formed colour product along 120 minutes of reaction time by comparing the calibration curves slope at the different scanning times; the dark grey bars represent the scanning time with calibration curve linearity is above 0.99, and the white bars represent the scanning time with calibration curves linearity below 0.99. The dark line represents the intercept of the calibration curves for each reaction time.
